# The Role of Natural Killer Cells in Soft Tissue Sarcoma: Prospects for Immunotherapy

**DOI:** 10.3390/cancers13153865

**Published:** 2021-07-31

**Authors:** Tânia Fortes-Andrade, Jani Sofia Almeida, Luana Madalena Sousa, Manuel Santos-Rosa, Paulo Freitas-Tavares, José Manuel Casanova, Paulo Rodrigues-Santos

**Affiliations:** 1 Center for Neuroscience and Cell Biology (CNC), Laboratory of Immunology and Oncology, University of Coimbra, 3004-504 Coimbra, Portugal; tania.andrade@student.uc.pt (T.F.-A.); jani.almeida@student.uc.pt (J.S.A.); luana.sousa@student.uc.pt (L.M.S.); 2 Faculty of Medicine, Immunology Institute, University of Coimbra, 3004-504 Coimbra, Portugal; msrosa@fmed.uc.pt; 3 Center of Investigation in Environment, Genetics and Oncobiology (CIMAGO), Faculty of Medicine, University of Coimbra, 3000-548 Coimbra, Portugal; jmcasanova@fmed.uc.pt; 4 Faculty of Medicine, Coimbra Institute for Clinical and Biomedical Research (iCBR), University of Coimbra, 3000-548 Coimbra, Portugal; 5 Center for Innovation in Biomedicine and Biotechnology (CIBB), University of Coimbra, 3000-548 Coimbra, Portugal; 6 Coimbra Hospital and University Center (CHUC), Tumor Unit of the Locomotor Apparatus (UTAL), University Clinic of Orthopedics, Orthopedics Service, 3000-075 Coimbra, Portugal; pftavares@chuc.min-saude.pt

**Keywords:** soft tissue sarcoma, natural killer cells, innate immunity, innate lymphoid cells, immunotherapy, immune checkpoint inhibitors, clinical trials

## Abstract

**Simple Summary:**

Soft-tissue sarcomas (STS) represent about 80% of sarcomas, and are a heterogeneous group of rare and malignant tumors. Morphological evaluation has been the standard model for the diagnosis of sarcomas, and even in samples with similar characteristics, they present genetic differences, which further increases the diversity of sarcomas. This variety is one of the main challenges for the classification and understanding of STS patterns, as well as for the respective treatments, which further decreases patient survival (<5 years). Natural Killer (NK) cells have a fundamental role in the control and immune surveillance of cancer development, progression and metastases. Notwithstanding the scarcity of studies to characterize NK cells in STS, it is noteworthy that the progression of these malignancies is associated with altered NK cells. These findings support the additional need to explore NK cell-based immunotherapy in STS; some clinical trials, although very tentatively, are already underway.

**Abstract:**

Soft-tissue sarcomas (STS) represent about 80% of sarcomas, and are a heterogeneous group of rare and malignant tumors. STS arise from mesenchymal tissues and can grow into structures such as adipose tissue, muscles, nervous tissue and blood vessels. Morphological evaluation has been the standard model for the diagnosis of sarcomas, and even in samples with similar characteristics, they present a diversity in cytogenetic and genetic sequence alterations, which further increases the diversity of sarcomas. This variety is one of the main challenges for the classification and understanding of STS patterns, as well as for their respective treatments, which further decreases patient survival (<5 years). Despite some studies, little is known about the immunological profile of STS. As for the immunological profile of STS in relation to NK cells, there is also a shortage of studies. Observations made in solid tumors show that the infiltration of NK cells in tumors is associated with a good prognosis of the disease. Notwithstanding the scarcity of studies to characterize NK cells, their receptors, and ligands in STS, it is noteworthy that the progression of these malignancies is associated with altered NK phenotypes. Despite the scarcity of information on the function of NK cells, their phenotypes and their regulatory pathways in STS, the findings of this study support the additional need to explore NK cell-based immunotherapy in STS further. Some clinical trials, very tentatively, are already underway. STS clinical trials are still the basis for adoptive NK-cell and cytokine-based therapy.

## 1. Introduction

Natural Killer (NK) cells are immune innate lymphoid cells (ILC) with a cytotoxic capacity for eliminating infected and transformed cells without restriction by the major histocompatibility complex (MHC), and they represent 5–15% of peripheral blood mononuclear cells. NK cells have a fundamental role in the elimination of infectious agents such as viruses, and in the control and immune surveillance of cancer development, progression and metastases [1].

The innate capacity of NK cells to identify and destroy cancer cells without MHC restriction has made them a promising subject for study in immunotherapy. The immune surveillance is mediated by a group of activatory and inhibitory receptors, the production of cytotoxic molecules such as granzyme B and perforin, and their regulation with other immune cells by cytokine and chemokine interaction [2,3,4].

Stressed or transformed cells express ligands that are not usually found in healthy tissue. Activatory NK cell receptors bind to these ligands and subsequently shift the balance to the direction of activation [5]. On the other hand, killer cell immunoglobulin-like receptors (KIRs) bind to human leukocyte antigens (HLA), which are expressed in all healthy cells and mediate inhibition signals [6].

The ability of NK cells to recognize and destroy circulating cancer cells is important for the control of metastases [7]. The capacity of autologous NK cells to infiltrate and lyse solid tumor cells is frequently deficient. Several mechanisms are associated with the loss of NK cell function and the appearance of cancer [8].

The study of the biology of NK cells in the last decade, namely their inhibitory and activatory receptors, and their respective ligands, has enabled us to understand the function of NK cells in several types of cancer, allowing the development of new strategies in cancer immunotherapy [9]. Advances in immunotherapy include strategies directed to immune checkpoint blockade, inhibiting the negative regulation of T cell activation and, more recently, NK cell-checkpoint blockade, such as the monoclonal antibodies (mAb) anti-KIR and anti-NKG2A [10]. Other recent immunotherapy strategies based on NK cells include cytokine-based therapy, adoptive NK-cell therapy and genetically modified NK cell therapy [11].

Soft tissue sarcomas (STS) are a group of rare and heterogeneous malignant tumors that could arise from mesenchymal tissues and can grow into structures such as adipose tissue, muscles, nervous tissue and blood vessels [12]. Currently, more than 50 different histological subtypes of STS have been described. The treatment for STS includes surgery, radiotherapy, chemotherapy and, more recently, immunotherapy.

Immunotherapy based on NK cells is still very recent, and the clinical trials are still mainly focused on hematological tumors and rarely some solid tumors. In this review, we summarize the biology of NK cells, their role in STS, as well as the prospects for the application of NK cell-based therapies in this group of cancers.

## 2. NK Cell Biology

Natural Killer (NK) cells are in the first line of defense against infections and tumor cells. They are derived from hematopoietic stem cells (HSC) of which the differentiation is induced by cytokines and transcription factors. Like other ILCs, NK cells derive from a common innate lymphoid precursor (CILP). ILCs were most recently discovered and classified into ILC1, ILC2 and ILC3 [13]. Despite the NK cells being a prototypical member of the group 1 ILCs, they have a distinct lineage and functionality [14]. NK cells are characterized by the expression of the T-box transcription factor 21 (TBX21), and by the production of IFN-γ, as well as the remaining ILC1 [15]; however, their differentiation from the others is manifested by the expression of Eomesodermin (EOMES) and cytolysis activity in response to infected or transformed cells, and the expression of MHC class I, NKp80 and/or the CD16 receptor [3].

Given the functional diversity in the expression of NK cell receptors in humans, it is difficult to characterize these cells. They are currently classified as CD3^−^, and by the expression of CD56 and/or CD16 distinguished into three subsets: CD56^bright/^CD16^−^, CD56^dim^/CD16^+^ and CD56^−^/16^+^ NK cells. CD56^bright^/CD16^−^ NK cells are classified as immature, with a high production of cytokines such as IFN-γ and TNF-α. CD56^dim^/CD16^+^ NK cells are considered mature cells with a high cytolytic capacity that express high levels of cytolytic granules containing granzyme B and perforin, inducing apoptosis by the Fas ligand and TNF-related apoptosis-inducing ligand (TRAIL) pathways. CD56^−^/16^+^ NK cells are characterized as being dysfunctional and associated with several pathological clinical conditions [16,17]. Initially, it was thought that the production of cytokines was restricted to CD56^bright^ NK cells, but nowadays it is known that CD56^dim^ NK cells produce a high amount of IFN-γ when they are activated [18].

Initially, it was thought that the bone marrow was the only place of maturation and differentiation of NK cells, but today it is recognized that immature NK cells can traffic and reside in several organs and tissue, where they can differentiate into phenotypically and functionally distinct mature cells [19,20]. In a recent study, Dogra et al. demonstrated that CD56^bright^/CD16^−^ NK cells reside predominantly in the lymph nodes, tonsils and gut, whereas CD56^dim^/CD16^+^ NK cells are mostly in the bone marrow (BM), peripheral blood (PB), spleen and lung. They observed that the greater the amount of NK cells in the BM, the larger the quantity of these in the PB [21].

The cytolysis and killing ability of NK cells are associated with the recognition of the target cells lacking the expression of Major Histocompatibility Complex (MHC) class I molecules. The opposite was proved by the hypothesis raised by Ljünggren and Kärre, where they demonstrated that cells that express MHC class I surface molecules are not lysed by NK cells after recognition, a process known as “missing-self” [6].

Currently, it is known that the function of NK cells is not only associated with their catalytic capacity but also with their interaction with other cells such as monocytes/macrophages and dendritic cells (DC) in mediating immune responses [22,23,24]. Depending on the state of maturation and the stimulus, NK cells express a differentiated repertoire of receptors that determine the state of activation or inactivation. Under normal physiological conditions, NK cells remain in a state of inactivation due to the recognition of MHC-I molecules (missing-self) from autologous cells by the expression of inactivation receptors such as: inhibitory Killer Ig-like receptors (iKIR); the C-type lectin receptor NKG2A, a member of CD94/NKG2 receptors; and the Leukocyte Ig-like receptor (LIRs/ILTs). KIRs were the first receptors to be identified, and they belong to the Ig subfamily, of which they recognize haplotypes of the HLA-A, HLA-B or HLA-C group. NKG2A recognizes HLA-E molecules, and LIRs recognize a variety of HLA Class I molecules [25,26]. These inhibitory receptors share common cytoplasmic domains named Tyrosine-based inhibition motifs immunoreceptor (ITIM) that allows the inactivation of NK cells when they recognize HLA class I molecules, which prevents the destruction of autologous cells. ITIM-based inhibition is dominant over activation in NK cells. The recruitment of SHP-1 by ITIM-bearing receptors inhibits signaling at a proximal step, in such a way that most downstream signals are blocked [27].

Regarding the expression of these receptors during the maturation of peripheral NK cells, it is known that the CD94/NKG2A heterodimer is expressed in the early stages (CD56^bright^), while KIRs emerge in the later stages (CD56^dim^), with the possibility of the co-expression of both in some transitional stages of maturation [28]. The expression of KIR or CD94/NKG2A in the different stages of maturation of NK cells is extremely important to maintain the state of inactivation by the recognition of HLA-class I antigens, which do not become autoreactive as the result of the process of the “licensing” or “education” of NK cells during the maturation phases. Unlicensed cells remain anergic, and are few in peripheral blood [29,30]. Currently, other non-specific HLA-class I inhibitory receptors have been identified as regulators of NK cell cytotoxicity, namely: the T cell immunoreceptor with Ig and ITIM domains (TIGIT), the PVR-related Ig domain receptor (PVRIG, also named CD112R), lymphocyte-activation gene 3 (LAG-3), T cell immunoglobulin and mucin domain containing 3 (TIM-3), programmed cell death protein 1 (PD-1), and T cell activation and increased late expression (TACTILE/CD96) [31].

**Figure 1:**
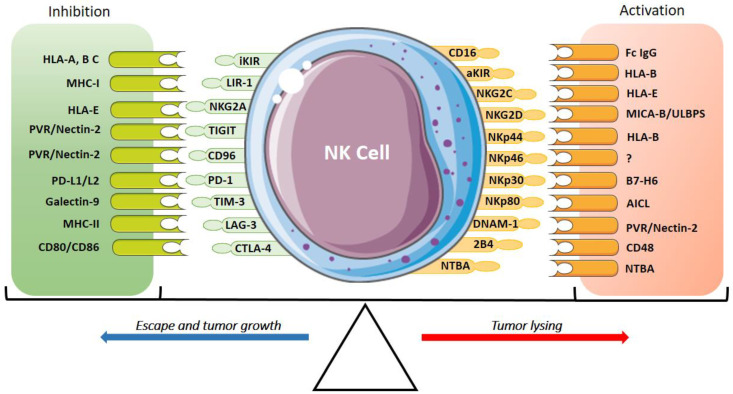
Inhibitory and activatory receptors of NK cells and their ligands. The expression of activatory receptors (CD16, aKIRs, NKG2C, NKG2D, NKp30, NKp46, NKp44 and NKp80), co-activator receptors (DNAM-1, 2B4, NTBA) and their ligands activate the cytotoxic response and tumor lysis of NK cells. The expression of inhibitory receptors (iKIR, NKG2A, TIGIT, TACTILE/CD96, TIM-3, LAG-3, CTLA-4, PD-1 and PVRG) suppresses the NK cell response promoting tumor growth. The activation and the inhibition are determined by the interaction of these receptors and their ligands.

In contrast, when there is a change in the target cells that does not allow “missing-self” recognition, such as in the case of infected or tumor cells, there is a positive regulation with the NK cell activation receptors to trigger the lytic mechanisms of killing target cells (non-self). This immunological regulation of NK cell activation is determined by the balance between the receptors and their respective ligands, which depends not only on the inactivation of the inhibitory receptors but also on the activation receptors and their respective ligands (Figure 1) [6,32].

A set of receptors capable of inducing NK cell activation for the detection and killing of target cells, namely NKp46, NKp44, NKp30, which are encoded by the genes NCR1, NCR2 and NCR3, respectively, have been described [33,34,35]. Despite structural and molecular differences, these receptors were collectively called “Natural Cytotoxicity Receptors” (NCR) [36]. NKp46 and NKp30 are expressed constitutively both in CD56^bright^ and CD56^dim^ NK cells, whereas NKp44 is expressed in subsets of CD56^bright^ NK cells. These receptors are present in other cells, namely ILCs and γδ T cells, and are activated by various viral, bacterial, fungal and lymphoma ligands, resulting in the release of IFN-γ and TNF-α [34,37].

NKG2D is another receptor for the activation of NK cell cytotoxicity activity identified in the recognition of damaged, infected, or transformed cells. It is a C-type lectin receptor and is a member of the NKG2 family, also expressed in some T cell subsets such as γδ T cells, CD8 T cells and some autoreactive or immunosuppressive CD4 T cells. It recognizes a variety of MHC class I ligands, such as the MHC class I chain-related protein (MICA/B) and the UL16 binding protein (ULBP1-6), which leads to signal transduction by this receiver [38].

Although NCRs and NKG2D play the central role in NK cell cytotoxicity, they can also work with co-receptors, enhancing the cytotoxicity of NK cells. Some of the co-receptors described are 2B4 [39], NTBA [40,41], DNAM-1 [42,43], CD59 [44] and NKp80 [45]. Activating receptors such as 2B4, NTBA, DNAM-1, NKG2D and NKp80 can be expressed at various stages of maturation, and are not restricted to a single NK cell subset [17,28]. The 2B4 co-receptor, despite having an activating function in mature NK cells, exhibits an inhibitory activity in the early stages of NK cell maturation, an effect resulting from the late appearance of SLAM-associated protein (SAP) [39,46].

In addition to inhibitory KIRs, activating KIRs that recognize HLA-C have also been described. Initially denominated as p50, these receptors have a homology on the outer portion with the p58 (inhibitory KIR), but differ in transmembrane and cytoplasmic structures [16,47,48]. The Toll-like receptors (TLR) responsible for recognition in innate immune cells have subsequently been identified in NK cells, playing a role in the combat of viral, bacterial and fungal infections [49,50]. Another activation receptor is HLA-E-specific NKG2C, expressed only in the final stages of maturation of NK cells, which is associated with cytomegalovirus (CMV) infection [51].

## 3. NK Cells in Cancer Disease

Up until now, it has been observed that the immune surveillance of NK cells is mediated by a set of receptors (activators and inhibitors) and their respective ligands, as well as their interaction with other immune cells. The hypothesis of “missing self” proposed by Ljünggren and Kärre made it possible to understand the mechanism by which NK cells kill transformed cells and spare normal cells, but it is still a great challenge for immunology to understand the escape mechanisms of this immunosurveillance, particularly in the case of tumor cells [6]. NK cells play a fundamental role in the control and development of cancer in the early stages, but can favor the progression of the disease in advanced stages of tumor transformation [7].

Currently, it is known that tumors develop mechanisms in the tumor microenvironment (TME) to escape the elimination of the immune system [52]. One of the escape mechanisms of tumor cells from immunosurveillance is the abnormal expression of MHC-I levels and the alteration of the TME that suppresses/inhibits the immune response, resulting in the progression of the cancer. [53]

NK cells can become cytotoxic to tumor cells and recruit other cells to TME by secreting pro-inflammatory cytokines and chemokines.

These interactions of NK cells with other cells have been one of the main challenges to understanding cancer progression and metastases [7]. Böttcher et al. demonstrated that the reduction of NK cells in the TME resulted in the failure of the recruitment of CD103^+^ DCs and immunological evasion by tumors in mice [54]. The arrival of NK cells in the TME leads to the recruitment of DCs by the secretion of chemokines, and the resident DCs culminate in the recruitment of CD8 T cells to the TME for the eventual destruction of the tumor [55]. The presence of monocytes/macrophages in primary tumors that produce IL-15 have also been proven to be important in the activation of NK cells with a production of IFN-γ, granzyme B and perforin, inhibiting the formation of metastases [56].

The new cancer treatment strategies focus on the development of immunotherapies based on immune checkpoint blockades. These therapies use mAbs blocking the inhibitory pathways in the negative regulation of T cell activation. However, the recent use of mAbs for the immune checkpoints of NK cells has gained space in the treatment of cancer. Anti-KIR and anti-NKG2A mAbs have been used as signal blockers to restore the anti-tumor cytotoxic activity of NK cells [10,57]. NK cells can also kill tumor cells via the low affinity FcγRIII (CD16) receptors by recognizing the specific IgG antibody through the antibody-dependent cellular cytotoxicity (ADCC) process [58].

Other immune checkpoints, notably co-inhibitory receptors such as CTLA-4 and PD-1, initially identified in T lymphocytes, have ligands for a variety of tumor-expressed molecules that induce an immunosuppressive response. More recently, it has been discovered that NK cells can also express these receptors, and can affect cytotoxic functions in tumor diseases [59]. PD-1 binds to its ligands, PD-L1 (CD274, B7-H1) and PD-L2 (CD273, B7-DC), and an increased expression of PD-1 ligands has been observed in several types of tumors after exposure to inflammatory cytokines or the activation of oncogenic pathways [59,60,61]. CTLA-4, the first immune checkpoint receptor to be clinically targeted, shares the same ligands, the B7 family (CD80 and CD86), with CD28, which are important to increase T and NK cell tolerance, but which also contributes to an immunosuppressive microenvironment in cancer [62].

Other receptors have been identified as immune checkpoints expressed in NK cells, such as TIGIT, TACTILE/CD96, LAG-3 and TIM-3 [63,64]. HLA class II molecules, expressed by antigen-presenting cells (APCs) and some tumor cells, are ligands of the LAG-3 receptor, making them attractive targets for immunotherapy. It has been suggested that another ligand for LAG-3, LSECtin, a member of the DC-SING family, is expressed in the liver and many tumors [65].

TIM-3 has several ligands: galactin-9 (Gal-9), phosphatidylserine (PtdSer), high mobility group protein B1 (HMGB1) and the carcinoembryonic antigen-related cell adhesion molecule 1 (CEACAM-1) [64]. TIGIT and TACTILE/CD96 are inhibitory co-receptors expressed by both T and NK cells, and are linked to the CD112 (Nectin-2) and CD155 (PVR) expressed by APCs, infected cells and tumor cells. In NK cells, the recognition of these ligands by TIGIT has an inhibitory effect by decreasing the production of IFN-γ and cytotoxic activity [63,66]. In contrast, the recognition of these ligands (CD112 and CD155) by DNAM-1 increases the NK cell-mediated cytotoxicity. Another recently identified inhibitory co-receptor is PVRIG, which recognizes CD112 [63]. NKLRB1 (CD161), a lectin C-type inhibitory receptor, has also been considered as a possible immune checkpoint, as it recognizes the lectin-like transcript 1 (LLT1) that is expressed by some tumors, such as B cell non-Hodgkin lymphoma. The positive regulation of this receptor has decreased NK cell cytolytic activity [67]. The analysis of these receptors has opened doors for the understanding of the activity of cells in the tumor microenvironment, as well as the new therapeutic possibilities for cancer.

Despite the growing evidence of the role of NK cells and their receptors in the immunosurveillance of tumors and in the prevention of metastases, it has been observed that there are flaws in this process that allow cancer to establish itself and progress. In fact, it has been seen that there are phenotypic changes in NK cells, and immunosuppressive factors in the TME such as a high expression of inhibitory ligands which favor tumor evasion and progression. TGF-β, for example, is associated with cancer progression by suppressing NKG2D expression, decreasing the NK cell cytotoxicity and IFN-γ production [68,69,70,71]. In addition to TGF- β, the secretion of other cytokines such as IL-10 by myeloid-derived suppressor cells (MDSC) and regulatory T cells (Treg) in TME raise the cytotoxic activity of NK cells, dendritic cells and macrophages [72].

The expression of the inhibitory KIR family (KIR2DL1, KIR2DL3, KIR2DL4 and KIR3DL1) as well as the reduced expression of activating receptors such as DNAM-1, NKG2C, NKp46 and NKp30, are also associated with poor cancer prognosis [31]. B7-H6, a ligand of NKp30, which is an activatory receptor of NK cells, appears to have a negative effect when expressed in high amounts by tumor cells. A study of patients with ovarian carcinoma has shown a loss of expression of the activating receptor NKp30 in tumor-associated NK cells, and this decrease has been associated with the presence of B7-H6, both in the soluble form and on the surface of the tumor cells [73].

Patients with acute myeloid leukemia (AML) showed phenotypic changes in NK cells when compared to healthy controls. These patients presented a downregulation of the NKp46 receptor and an overexpression of NKG2A, with significantly reduced CD107a degranulation and IFN-γ and TNF-α production [74]. The reduced expression of DNAM-1 in patients with solid cancer and leukemia may decrease the NK cell-mediated cytotoxicity by the positive regulation of TIGIT or PVRIG inhibitory receptors [63].

Several mechanisms are associated with the loss of NK cell function and the appearance of cancer. It is known that decreased NK cell cytotoxicity is associated with the expression and/or the functionality of defective NK cell activating receptors in the elderly [75,76]. Alterations in the phenotype are one of the main characteristics associated with NK dysfunction in several hematological cancer and solid tumors [4]. The effect of both age and cancer may act synergistically to downregulate the NK cell-mediated tumor immunosurveillance. NK cells from acute myeloid leukemia (AML) patients show a diminished expression of several activatory receptors that contribute to impaired NK cell function [77,78].

Gounder et al. examined the age-associated changes in the NK cell population and their subsets in healthy donors in different age groups of males and females (41 to 80 years) [79]. The data showed that the level of total immune cells also dropped on aging. However, the total NK cell population was remarkably increased, with the majority of NK cells being CD56^dim^. The evaluation of the proliferation potential of NK cells showed that NK cell proliferation ability declines with age. These results suggest that there is an increase in the circulating NK cell population upon aging; however, the proliferation rate decreases with aging.

## 4. NK Cells in Soft Tissue Sarcomas

Soft tissue sarcomas (STS) represent about 80% of sarcomas, and are a heterogeneous group of rare and malignant tumors arising in connective tissues embryologically derived from the mesenchyme [80]. Based on their histological and molecular characteristics, the STS can be divided into several subtypes. Morphological evaluation has been the standard model for the diagnosis of sarcomas, and even in samples with similar characteristics, they present a diversity in their cytogenetic and genetic sequence alterations, which further increases the diversity of sarcomas. This variety is one of the main challenges for the classification and understanding of STS patterns, as well as for the respective treatments, which further decreases patient survival (<5 years) [81,82].

The last classification of STS was made by the World Health Organization (WHO) in 2013 [68]. According to the WHO classification, these tumors are usually located in the extremities, trunk wall and retroperitoneum, but not in internal organs (visceral sarcomas). As for the CONTICANET and RARECARE projects, the most frequent histotypes of STS were liposarcoma, leiomyosarcoma and dermatofibrosarcoma protuberans, and angiosarcoma [83,84]. A large proportion of leiomyosarcoma in females are located in the uterus [83]. The Reference Network for Pathology of Soft Tissue-GIST-Desmoid-Visceral Sarcomas (RRePS) conduced a histopathological review study of sarcoma, and estimated that the prevalent STS histotypes in France are: liposarcoma, undifferentiated pleomorphic sarcoma, angiosarcoma, rhabdomyosarcoma, synovial sarcoma, and malignant tumors of the sheath peripheral neural [85].

STS can be diagnosed in all age groups, although they are more frequent in adults over 50 years old, with an average age of diagnosis of 58 years. Although sarcomas are mostly diagnosed in adults, rhabdomyosarcoma and Ewing sarcoma are more common in the younger population (children and adolescents) [81,84]. Some studies have shown that the most common STS are diagnosed in the 40–60 age group, with an average of 60 years (for male and females); however, they can be diagnosed in the older population (see Table S1). Given that STS are mostly diagnosed in the older adult population, studies to evaluate the effect of age on this disease group is important. Now, it’s known that aging is associated with the emergence of cancer diseases and alterations in the immunological profile. Rodrigues-Santos et al. evaluated the effect of age on NK cells in chronic myeloid leukemia (CML) patients treated with Tyrosine Kinase inhibitors (TKIs) [86]. Significant differences of the phenotype and function of NK cells were found between middle-aged (35–65 years old) and elderly (older than 65) patients and healthy individuals.

Despite some studies, little is known about the immunological profile of STS. An immunohistochemistry survey, carried out by D’Angelo et al. on 50 samples from STS patients, evaluated the expression of PD-L1 and the quantification of tumor-infiltrating lymphocytes (TIL) [87]. These authors analyzed the PD-L1 expression of tumors, lymphocytes and macrophages, with a result of 12%, 30% and 58%, respectively, with a higher prevalence in gastrointestinal stromal tumors (GIST). They observed an infiltration of lymphocytes and macrophages in 98% and 90% of the samples, respectively, and there was no association between the clinical characteristics, survival and expression of PD-L1. Another study, conducted by Movva et al., also evaluated the expression of PD-1 and and PD-L1 in sarcomas by immunohistochemistry [88]. The significant expression of PD-L1 was seen in leiomyosarcoma (32%), chondrosarcoma (75%), liposarcoma (77%) and undifferentiated pleomorphic sarcoma (70%).

As for the immunological profile of the STS related to NK cells, there is also a shortage of studies. Observations made in solid tumors show that the infiltration of NK cells into tumors is associated with a good prognosis of the disease [89]. Torabi et al. evaluated the expression of PD-1 and PD-L1 in STS and bone sarcomas. Tissue microarrays for liposarcomas, rhabdomyosarcomas, conventional osteosarcomas and chondrosarcomas were stained for PD-1 and PD-L1, and they observed that the expression of PD-1 in rhabdomyosarcomas was associated with a more progressed stage of the tumor [90]. The results also showed that one case of pleomorphic liposarcoma, one case of pleomorphic rhabdomyosarcoma and two cases of alveolar rhabdomyosarcoma were positive for PD-L1. In another study by Zhang et al., three osteosarcoma cell lines were used to evaluate the susceptibility of these cell lines to the cytolytic activity from isolated health donor NK cells [91]. In addition, they analyzed the expression of PD-L1 in cell lines using anti-PD-L1 mAb. These results suggest that the PD-1/PD-L1 axis plays an important role in the cytotoxicity of NK cells through granzyme B secretion.

The expression of NK cell receptors is important for the progression of cancer. Despite the scarcity of studies to characterize NK cells, their receptors and ligands in STS, it is noteworthy that the progression of these malignancies is associated with altered NK phenotypes. For example, Delahaye et al. showed that the NKp30 receptor, involved in the recognition of tumor cells and DCs, is involved in the prognosis of GIST [92]. They observed that healthy individuals had different NKp30 isoforms from those of patients with GIST. They also observed that the expression of the immunosuppressive isoform (NKp30c) was superior to the expression of the immunostimulatory isoforms (NKp30a and NKp30b), and it was also associated with a lower survival of patients with GIST.

The tumor microenvironment greatly affects the normal expression of NK cell receptors, leading to a decrease in activatory receptors and an increased expression of inhibitory receptors, affecting the balance in the cytotoxic response. Verhoeven et al. observed that Ewing sarcoma cell lines and primary Ewing sarcoma tumor cells expressed ligands for the activatory NK cell receptors NKG2D and DNAM-1, and that the NK cell cytotoxicity to Ewing sarcoma cells critically depended on the combination of NKG2D and DNAM-1 signaling, [93]. Thus, they observed that the blockade of one of these receptors abolished lysis by resting NK cells, and that the cytotoxicity of NK cells in patients with EWS was reduced in comparison with healthy individuals (controls of the same age).

Another preclinical study investigated the potential of NK cells, at rest and activated by cytokines, to lyse RMS cell lines, as well as the pathways involved [94]. The cell lines were susceptible to NK cell-mediated cytolysis at rest, and this susceptibility was significantly increased using IL-15-activated NK cells. Flow cytometry and cytolytic assays revealed that the receptor–ligand interactions NKG2D and DNAM-1 were essential in cytolysis for resting NK cells, as the simultaneous blocking of both pathways resulted in the almost-complete revocation of cytotoxicity. On the other hand, the combined blocking of DNAM-1 and NKG2D only led to a partial reduction in the lytic activity of IL-15-activated NK cells. In this regard, the residual lysis was at least in part mediated by pathways involving the natural cytotoxicity receptors NKp30 and NKp46.

Borowski et al. examined the expression of polymorphic and non-polymorphic MHC antigens in Ewing tumor cells, and observed that the exceptionally constant expression of HLA-C or some other non-A and non-B antigens (reactive with defined monoclonal antibodies) has important consequences for the resistance of tumor cells against specific CTL and NK cell activity in vivo [95].

## 5. NK Cell and Immunotherapy in Soft Tissue Sarcoma

In many cases, the lack of information regarding molecular signatures for STS means that treatments are still based on surgery/resection, radiation and/or conventional chemotherapy. Some of these treatments for STS, such as gastrointestinal sarcomas and leiomyosarcoma, have been based on TKI—such as imatinib, or sunitinib and regorafenib—in cases of progression and metastases. Another TKI used in the treatment of STS is pazopanib, and is a target of vascular endothelial growth factor receptors (VEGFR) and platelet-derived growth factor receptors (PDGFR). This drug has been shown to be favorable in some clinical trials [96,97]. Olaratumab, a mAb against PDGFRa, used in conjunction with doxorubicin in a phase II clinical trial published by Lancet in 2016, seemed promising in STS treatment. However, the results were not as expected in phase III, which led the Food and Drug Administration (FDA) to remove olaratumab from STS treatment in 2019 [98].

Today, new research strategies based on immunotherapy are being considered, which allow a longer survival and better quality of life for patients with STS. Some of these therapies include: chimeric antigen receptor (CAR) T cells, recombinant IFN-γ, the use of monoclonal antibodies (e.g., PDL-1 and CTLA-4), lymphocyte transfer and autologous DCs [99].

Some immunotherapy strategies based on NK cells have been developed, namely the stimulation of NK cells with cytokine-based therapy, antibody-based therapy, NK cell adoptive therapy and genetically modified NK cell therapy (CAR-NK cells) [11].

### 5.1. Antibody-Based Immunotherapy

Specific antibodies are used to block inhibitory receptors or to engage activating receptors to improve NK cell responses (Table 1).

Tawbi et al., in a cohort study (SARC028), investigated the use of pembrolizumab (anti-PD-1) in 42 STS (leiomyosarcoma, dedifferentiated liposarcoma, undifferentiated pleomorphic sarcoma and synovial sarcoma) and bone sarcoma [100]. In general, pembrolizumab has not been shown to be effective (especially in leiomyosarcoma and liposarcoma), but it has shown a stimulating activity in patients with de-differentiated liposarcoma (NCT02301039 in clinicaltrials.gov/, accessed on 31 May 2021) [84]. A case study involving a 63-year-old patient proved that using pembrolizumab in the treatment of angiosarcoma could be effective [101].

D’Angelo et al. reported a randomized study in unresponsive and metastatic patients with advanced sarcomas where they used nivolumab (anti-PD-1) alone or in combination with ipilimumab (anti-CTLA) [85]. They observed that nivolumab alone had effective results for sarcomas, and that—in combination with ipilimumab—there is promising efficacy for some sarcoma subtypes. Ben-Ami et al. reported a study of nivolumab in advanced uterine leiomyosarcoma but no responses were reported, and the study closed early because of a lack of efficacy [103]. Maki et al. investigated the clinical activity of single agent anti-CTLA4 antibody ipilimumab in patients with advanced or metastatic synovial sarcoma [106]. The patients were treated with ipilimumab 3 mg/kg intravenously every 3 weeks for three cycles, and then restaged. All of the patients showed clinical or radiological evidence of disease progression after no more than three cycles of therapy, for a Response Evaluation Criteria in Solid Tumors (RECIST) response rate of 0%. The study was stopped for slow accrual, lack of activity and a lack of immune response.

It seems that the use of anti-CTLA4 alone does not result in good clinical responses. However, in combination therapy with anti-PD-1, the best therapeutic responses are obtained. In addition, the combined use of ICIs with chemotherapeutics may result in better immunoregulatory responses in STS.

Toulemonde et al., in a phase two clinical trial, assessed the efficacy and safety of PD-1 targeting (pembrolizumab) in combination with metronomic chemotherapy in sarcomas [104]. This phase two study of four cohorts of patients with advanced STS included leiomyosarcoma, undifferentiated pleomorphic sarcoma, other sarcomas, and GIST. They concluded that PD-1 inhibition has limited activity in selected STS and GIST. This may be explained by an immunosuppressive tumor microenvironment resulting from macrophage infiltration and IDO1 pathway activation. Another study evaluated the activity of the VEGFR using axitinib (TKI), plus pembrolizumab (anti-PD-1) in patients with sarcoma [105]. VEGF promotes an immunosuppressive microenvironment and contributes to ICIs resistance in cancer. These results showed that the axitinib plus pembrolizumab has manageable toxicity and preliminary activity in patients with advanced sarcomas, particularly patients with alveolar soft-part sarcoma, warranting further investigation in randomized controlled trials.

The use of anti PD-L1 mAb—such as avelumab, atezolizumab and durvalumab—has also been evaluated both with, or without, combined therapy in clinical trials on STS such as liposarcomas, leiomyosarcomas, Synovial sarcoma, angiosarcoma and Ewing sarcoma (NCT030074318, NCT02609984, NCT03111729, NCT030176529, NCT03111729, NCT0301765 in clinicaltrials.gov/, accessed on 31 May 2021). Other clinical trials using anti-PD-1 (nivolumab) and anti-CTLA4 (ipilimumab and tremelimumab) are in progress (NCT03463408, NCT03116529, NCT02815995, NCT03138161 in clinicaltrials.gov/, accessed on 31 May 2021).

**Figure 2:**
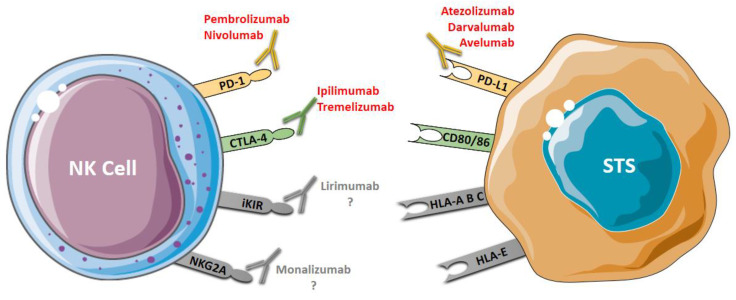
Antibody-based immunotherapy in STS. PD-1 and CTLA-4 are co-inhibitor receptors expressed by NK that can be blocked using immune checkpoint inhibitors: anti-PD-1 (pembrolizumab and nivolumab) and anti-CTLA-4 (ipilimumab and tremelizumab). PD-L1, a ligand of PD-1 expressed by tumor cells, can be blocked using anti-PD-L1 (avelumab, darvalumab e atezolizumab). iKIR and NKG2A, two specific NK inhibitory receptors, can be blocked by anti-KIR (lirimumab) and anti-NKG2A (monalizumab); however, there are no studies on the use of these drugs (lirimumab e monalizumab) in STS. Blocking these receptors restores the cytotoxic activity of the NK cells.

The use of mAbs that block specific inhibitory receptors of NK cells, such as lirimumab and monalizumab, has been evaluated in hematologic cancers and represents potential agents targeting NK cells for STS immunotherapy (Figure 2).

Lirilumab is a fully human IgG4 antibody targeting KIR2DL1, KIR2DL2, KIR2DL3, KIR2DS1 and KIR2DS2. This mAb has been shown to have therapeutic potential for acute myeloid leukemia, and in multiple myeloma [57,107]. Monalizumab, an IgG4 blocking mAb against NKG2A, is the first ICI that can target both T cell and NK cell responses. This mAb was designed to target receptors with ITIM domains, and it can improve the function of NK cells in combination with anti-PD-1 (durvalumab) or anti-EGFR (cetuximab) [10]. Another mAb used to increase NK cell-mediated antitumor activity is elotuzumab. Elotuzumab is a humanized antibody which can engage SLAMF7 on effector NK cells and directly increases their function. This antibody also coats SLAMF7 on specific cells and attracts CD16^+^ NK cells to exert NK cell-mediated ADCC. Continuously, the interaction between the SLAMF7 on NK cells and the SLAMF7 on target cells promotes the anti-cancerous effect of NK cells in a manner independent of ADCC [108].

### 5.2. NK Cell-Based Adoptive Therapy

The infusion of autologous lymphocytes and the use of immunostimulators has also gained ground in the treatment of some types of sarcomas. Patients with recurrent rhabdomyosarcoma and metastases treated with an infusion of autologous lymphocytes and IL-7 presented higher survival rates than those treated only with autologous lymphocytes [99]. Today, therapies based on NK cells have gained greater attention in cancer immunotherapy. The cytotoxic ability of NK cells to kill tumor cells without MHC (“non-missing self”) recognition has sparked interest in the study and development of NK cell-based immunotherapy [32]. Although NK cells are of interest for immunotherapy, some limitations—such as the level of the penetration of the NK cells into the tumor microenvironment—have been a major challenge in clinical trials.

One of the first therapies applied was the adoptive transfer of autologous or allogeneic NK cells obtained from peripheral blood. These cells are usually stimulated and expanded by cytokines, such as IL-2 or IL-15, or in tumor cell co-cultures (K562 cell line), and then injected into the patient. Despite the limited efficacy of this therapy, it has had some success in hematological malignancies, but it remains ineffective for solid tumors. This efficacy is related to the increase in Treg due to the administration of immunostimulators such as IL-2 [89].

NK cell-based immunotherapies are still limited to pre-clinical and clinical trials in hematological diseases and some solid tumors. STS clinical trials are still the basis for adoptive NK cell and cytokine-based therapy (see Table 2).

Cho et al. conducted a study to develop new therapies for pediatric cancers, and tested the cytotoxicity of expanded NK cells, stimulated by contact with K562-mb15-41BBL cells, to exert cytotoxicity against some sarcoma cell lines. Of the STS lines tested in vitro, Ewing’s sarcoma and rhabdomyosarcoma were more sensitive to the cytotoxicity of expanded NK cells [109]. This study led to a pilot clinical study in which they attested that the infusion of expanded, activated haploidentical NK cells can produce measurable clinical responses in patients with Ewing sarcoma and rhabdomyosarcoma (NCT02409576 in clinicaltrials.gov/, accessed on 31 May 2021). Currently, therapies based on NK cells applied in STS are limited to infusions of expanded NK cells and/or in combination with other therapies (See Table 2).

The injection of the NK-92 cell line has also been shown to be effective in the treatment of some tumors. The NK-92 cell line obtained from a patient with non-Hodgkin lymphoma of NK cells with a CD3^−^/CD56^+^/CD16^−^ immunophenotype retained cytotoxic antitumor activity. So far, there are no clinical trials using NK-92 cells in STS, but the intratumoral injection of NK-92 cells in refractory Ewing sarcoma has been proven to be safe, with no toxic responses and with preliminary evidence of response [110].

In addition to the expansion of NK cells, the modification of NK cell-associated genes has gained ground in adoptive immunotherapy to increase the efficiency and cytotoxicity of NK cells. It was demonstrated that NK cell lines modified for the IL-15 gene (NKL-IL15) showed greater cytolytic activity in hepatocellular carcinoma and leukemia [111,112]. This cytotoxicity was measured by the expression of cytolysis-related molecules such as NKp80, TRAIL, granzyme B, IFN-γ and TNF-α.

The use of an IL-15 super-agonist, ALT-803, also increased the functionality of NK cells against ovarian cancer cell lines with the significant expression of CD107a, IFN-γ and TNF-α [113]. The aim of a phase I clinical study, still in the early stages, is to test a combination of NK cell infusion therapy with the administration of the ALT-803 fusion protein in some tumors, including STS (NCT02890758 in clinicaltrials.gov/, accessed on 31 May 2021). In a phase one clinical study, 19% of the patients with hematologic cancers were observed to have responses after administration with ALT-803, including one complete remission lasting seven months [114]. In addition to peripheral blood NK cells, human embryonic stem cells, induced pluripotent stem cells (iPSCs) and bone marrow or umbilical cord blood are being studied, and represent potential alternative sources of therapeutic NK cells [115].

Recently, the development of CAR-T cells represented a breakthrough in immunotherapy against hematological neoplasms. However, there are still several obstacles, such as the occurrence of chronic cytotoxicity and graft-versus-host-disease (GVHD), that limited its clinical application. Another constraint is the fact that this therapy with autologous cells is very expensive [116]. Some clinical trials in progress already use engineered CAR-T cells in STS and Ewing sarcoma (NCT03960060, NCT00902044, NCT04433221 and NCT03635632 in clinicaltrials.gov/, accessed on 31 May 2021).

The use of CAR-NK cells seems to be safer than CAR-T because it is less cytotoxic and less likely to cause GVHD after allogeneic infusion. The short life of NK cells becomes an advantage for CAR-NK cell therapy, as it prevents chronic toxicity. They can be supplemented with IL-15 to increase the useful life of the system. Now, CAR-NK cells are being studied in clinical trials targeting hematological neoplasms and some solid tumors, but there is still no study on STS [117]. UCB-derived CAR-NK cells are being investigated in a phase I/II study for the treatment of refractory B-cell leukemia and lymphomas (NCT03056339 in clinicaltrials.gov/, accessed on 31 May 2021).

## 6. Conclusions and Future Directions

Understanding the biological mechanisms of NK cells and their interaction with other cells and immunoregulators has created new paths for the development of therapies based on NK cells in hematological and solid cancers. However, in rare cancers such as STS, there is still a long way to go. Despite the scarcity of information on the function of NK cells, their phenotypes and regulatory pathways in STS, these findings support the additional need to explore NK cell-based immunotherapy in STS. Some clinical trials, although only tentatively, are already underway.

The expression of PD-1 and PD-L1 in STS are associated with poor prognosis. ICIs have been widely used to block this pathway. In general, pembrolizumab has not been shown to be effective in some types of STS (especially in leiomyosarcoma and liposarcoma). However, it was observed that better results can be obtained when they are combined with chemotherapeutics. Ipilimumab showed better results when combined with nivolumab. These findings suggest that combination therapy remains one of the best strategies to boost immune responses and slow down the disease progression in STS. The use of anti-iKIR (lirilumab) and anti-NKG2A (monalizumab) has shown promising results in the treatment of hematologic cancers. As they are targets of specific NK cell receptors, studies to assess the use of these drugs in STS would be of great value. Other receptors have been identified as ICIs expressed by NK cells, such as TIGIT, TACTILE/CD96, LAG-3 and TIM-3, but there are still few studies of these receptors in STS.

The NK cell-based immunotherapies are still limited to pre-clinical and clinical trials in hematological diseases and some solid tumors. The expansion of NK cells and the modification of NK cell-associated genes have been used in adoptive immunotherapy to increase the efficiency and cytotoxicity of NK cells.

The growing investigation of the function of NK cells, their phenotypes and the regulatory pathways in STS, as well as the pioneer clinical trials described in this review, justifies further investment in developing NK cell-based immunotherapy strategies for STS.

## Figures and Tables

**Table 1:** Antibody-based immunotherapies in soft tissue sarcoma (completed clinical trials).

Drug	Target	Outcome (Descriptions)	Reference
Pembrolizumab	PD-1	Effective results in STS. Response: 18% in STS, 40% in undifferentiated pleomorphic sarcoma, 20% in liposarcoma, 10% in synovial sarcoma. Stimulating activity in dedifferentiated liposarcoma.	[100]
	PD-1	Effective in the treatment of angiosarcoma	[101]
Nivolumab + Ipilimumab	PD-1 CTLA-4	Nivolumab alone had effective results for sarcomas, but that in combination with ipilimumab there is promising efficacy for some sarcoma subtypes (nivolumab: 5%; ipilimumab plus nivolumab: 16%).	[102]
Nivolumab	PD-1	Did not demonstrate a benefit in this cohort of previously treated advanced uterine leiomyosarcoma patients.	[103]
Pembrolizumab + Metronomic cyclophosphamide	PD-1	Nonprogression and objective responses at 6 months per Response Evaluation Criteria in Solid Tumors (RECIST) for undifferentiated pleomorphic sarcoma, leiomyosarcoma and others, and GIST were 0%, 0%, 14.3% and 11.1%, respectively.	[104]
Pembrolizumab + Axitinib	PD-1 VEGFR	With a median follow-up of 14.7 months, 3-month progression-free survival for all evaluable patients was 65.6%. For patients with alveolar soft-part sarcoma, 3-month progression-free survival was 72.7%. Has manageable toxicity and preliminary activity in patients with advanced sarcomas.	[105]

**Table 2:** Completed and ongoing NK cell-based immunotherapies (ClinicalTrials.org, accessed on 31 May 2021) in soft tissue sarcomas.

Condition/Disease	Study/Title	Therapy	Phase	Trial ID
***Completed***				
Recurrent adult STS Others	Combination of cryosurgery and NK immunotherapy for recurrent sarcoma	Cryosurgery Multiple NK immunotherapies	I/II	NCT02849366
Rhabdomyosarcoma Others	NK DLI in Patients After Human Leukocyte Antigen (HLA)-Haploidentical Hematopoietic Stem Cell Transplantation (HSCT)	CD3-Depleted/CD56 + selected natural killer cells collected from apheresis products	I/II	NCT01386619
Sarcoma Others	NK White Blood Cells and Interleukin in Children and Young Adults With Advanced solid tumors	Recombinant human interleukin-15 (rhIL-15) NK Cell Infusion	I	NCT01875601
Ewing sarcoma Rhabdomyosarcoma	Pilot study of expanded, activated Haploidentical NK Cell infusions for sarcomas (NKEXPSARC)	Infusion of allogeneic NK + IL-2	I/II	NCT02409576
Ewing sarcoma Rhabdomyosarcoma STS	Haploidentical Stem Cell Transplantation and NK Cell Therapy in Patients With High-risk solid tumors	Haploidentic HSCT Expanded NK cell + IL-2	II	NCT01807468
***Ongoing***				
Ewing sarcoma Rhabdomyosarcoma	Phase I Trial of Universal Donor NK Cell Therapy in Combination with ALT803	NK cell ALT803 (IL-15 superagonist)	I	NCT02890758
Ewing sarcoma Rhabdomyosarcoma Others	Phase 2 STIR Trial: Haploidentical Transplant and Donor NK Cells for Solid Tumors	Allogeneic HSCT Donor NK Cell Infusion	II	NCT02100891
Recurrent and refractory malignant soft tissue neoplasm others	Ex-Vivo Expanded Allogeneic NK Cells For The Treatment Of Pediatric Solid tumors	Cord Blood-derived Expanded Allogeneic NK Cells Cyclophosphamide Etoposide	I	NCT03420963

## Supplementary Materials

The following are available online at https://www.mdpi.com/article/10.3390/cancers13153865/s1; Table S1: Age and most common soft tissue sarcomas histotypes.
