# Supplementary material for: The Role of Natural Killer Cells in Soft Tissue Sarcoma: Prospects for Immunotherapy
Source: Cancers (Basel). 2021 Jul 31;13(15):3865. doi: 10.3390/cancers13153865 (PMC8345358; doi:10.3390/cancers13153865)
Supplement: Supplementary file 1 [file cancers-13-03865-s001.zip › cancers-1263428-supplementary.pdf]

cancers

MDPI

Supplementary Material: The role of Natural Killer Cells in Soft Tissue Sarcoma: Prospects for Immunotherapy

Tânia Fortes-Andrade, Jani Sofia Almeida, Luana Madalena Sousa, Manuel Santos-Rosa, Paulo Freitas-Tavares, José Manuel Casanova and Paulo Rodrigues-Santos

Table S1. Age and most common soft tissue sarcomas histotypes.

STS Histotype	Median Age	Sex	Ref.
Liposarcoma	≥60 years	164/103 (M/F)	[1]
	60 years (median age for all STS)	Prevalent in male (all STS)	[2]
	60 years (median age for all STS)	Prevalent in female (all STS)	[3]
Leiomyosarcoma	≥40 years	84/168 (M/F)	[1]
	60 years (median age for all STS)	Prevalent in male (all STS)	[2]
	60 years (median age for all STS)	Prevalent in female (all STS)	[3]
	60 years	Prevalent in male (all STS)	[4]
Undifferentiated pleomorphic sarcoma	60 years (median age for all STS)	Prevalent in female (all STS)	[3]
	60 years (median age for all STS)	Prevalent in male (all STS)	[2]
Dermatofibrosarcoma protuberans	40-60 years	48/50 (M/F)	[1]
Rhabdomyosarcoma	0-20 and ≥80 years<br>Bimodal trend (a peak in childhood and a rapid increase in later life)	34/17 (M/F)	[1]
Angiosarcoma	≥80 years	20/41 (M/F)	[1]
Synovial sarcoma	-	17/17 (M/F)	[1]
	60 years (median age for all STS)	Prevalent in male (all STS)	[2]
Gastrointestinal stromal tumors (GIST)	≥70 years	180/183 (M/F)	[1]
Malignant Fibrous Histiocytoma/ Mixofibrosarcoma	60 years (median age for all STS)	Prevalent in male (all STS)	[4]
	60 years (median age for all STS)	Prevalent in male (all STS)	[2]
Complex Mixed and Stromal Neoplasms	60 (median age for all STS)	Prevalent in male (all STS)	[4]

References

1. Mastrangelo, G.; Coindre, J.-M.; Ducimetière, F.; Tos, A.D.; Fadda, E.; Blay, J.-Y.; Buja, A.; Fedeli, U.; Cegolon, L.; Frasson, A.; et al. Incidence of soft tissue sarcoma and beyond. *Cancer* **2012**, *118*, 5339–5348, doi:10.1002/cncr.27555.
2. Honoré, C.; Méeus, P.; Stoeckle, E.; Bonvalot, S. Soft tissue sarcoma in France in 2015: Epidemiology, classification and organization of clinical care. *J. Visc. Surg.* **2015**, *152*, 223–230, doi:10.1016/j.jviscsurg.2015.05.001.
3. Kollár, A.; Rothermundt, C.; Klenke, F.; Bode, B.; Baumhoer, D.; Arndt, V.; Feller, A. Incidence, mortality, and survival trends of soft tissue and bone sarcoma in Switzerland between 1996 and 2015. *Cancer Epidemiol.* **2019**, *63*, 101596, doi:10.1016/j.canep.2019.101596.
4. Bessen, T.; Caughey, E.G.; Shakib, S.; Potter, J.A.; Reid, J.; Farshid, G.; Roder, D.; Neuhaus, S.J. A population-based study of soft tissue sarcoma incidence and survival in Australia: An analysis of 26,970 cases. *Cancer Epidemiol.* **2019**, *63*, 101590, doi:10.1016/j.canep.2019.101590.
